# Letter to editor in response to Has *Chlamydia trachomatis* prevalence in young women in England, Scotland and Wales changed? Evidence from national probability surveys

**DOI:** 10.1017/S0950268819001572

**Published:** 2019-09-20

**Authors:** P. J. White, J. Lewis

**Affiliations:** 1 MRC Centre for Global Infectious Disease Analysis and NIHR Health Protection Research Unit in Modelling Methodology, Imperial College London, London, UK; 2 Modelling and Economics Unit, National Infection Service, Public Health England, London, UK

Robust assessments of chlamydia screening programme performance are urgently needed [1]. Unfortunately, after more than a decade of screening by England's National Chlamydia Screening Programme (NCSP), there is still not definitive evidence of effectiveness [2]. Kounali *et al*. propose using ‘serological markers… in… sentinel populations’ [3]. However, it is highly unlikely that serology will provide information at local level so analysis of routine surveillance data will still be required to monitor programme performance and address health inequalities, using methods such as our model, which estimates chlamydia incidence and prevalence by synthesising surveillance data with information on natural history and behavioural parameters [2, 4, 5].

Kounali *et al*. assert that our model assumes that ‘treatment-seeking and diagnosis-seeking behaviour has stayed constant’ [3], without defining their terms. Our model distinguishes ‘screening’ (testing in the absence of symptoms) from testing sought by symptomatic individuals. We have already pointed out in response to two of the authors [6] that the probability of being screened varies by place in our geographic analysis [4] and by time in our temporal analysis considering England as a whole [2]. There are no data to inform on any changes over time in the rate of testing prompted by symptoms. However, sensitivity analysis indicated that our prevalence estimates were insensitive to this parameter over a range of plausible values for England [4, 6].

Kounali *et al*. speculate that ‘awareness campaigns linked to the NCSP is likely to have increased diagnostic testing-seeking behaviour in asymptomatic women after a potential exposure’ [3], but provide no evidence. The study they cite did not assess any awareness campaign, and took place in Scotland – where the NCSP has never operated – during a 12–14 month period when the NCSP was only beginning to be implemented in some areas of England.

The statement that our results ‘point to little change in prevalence after 2000, until 2008–2010, which coincides with the date when the methods for recording coverage and diagnosis rates changed’ is incorrect. In fact, the incomplete data before 2008 cause large uncertainty, meaning that our estimates do not point to stable prevalence; rather, we are not able to estimate the magnitude and direction of prevalence changes precisely [2].

We are pleased that NCSP authors recognise the importance of understanding patient behaviour and reason for testing in interpreting surveillance data. We ourselves highlighted that our analysis is limited by the available data [2, 4–6], but it already incorporates data on health-seeking behaviour [4] and can readily use further data if collected. With more than a million tests per year, NCSP could collect a rich spatiotemporal data set. We hope that NCSP will henceforth collect the data we recommend: presence and duration of any symptoms, the patient's reason(s) for testing (e.g. symptoms, partner notification, perceived risk of recent exposure, retesting after a positive test), and associated sexual risk behaviour [2, 4, 6].

Furthermore, we recommend that the next National Survey of Sexual Attitudes and Lifestyles (http://www.natsal.ac.uk) population-based survey collects corresponding data to enable calculation of population-based rates, with numerator data coming from screening services and denominator data from the survey.
